# Supplementary figures and images for: Spatial changes in park visitation at the onset of the pandemic
Source: PLOS Glob Public Health. 2022 Sep 13;2(9):e0000766. doi: 10.1371/journal.pgph.0000766 (PMC10021367; doi:10.1371/journal.pgph.0000766)

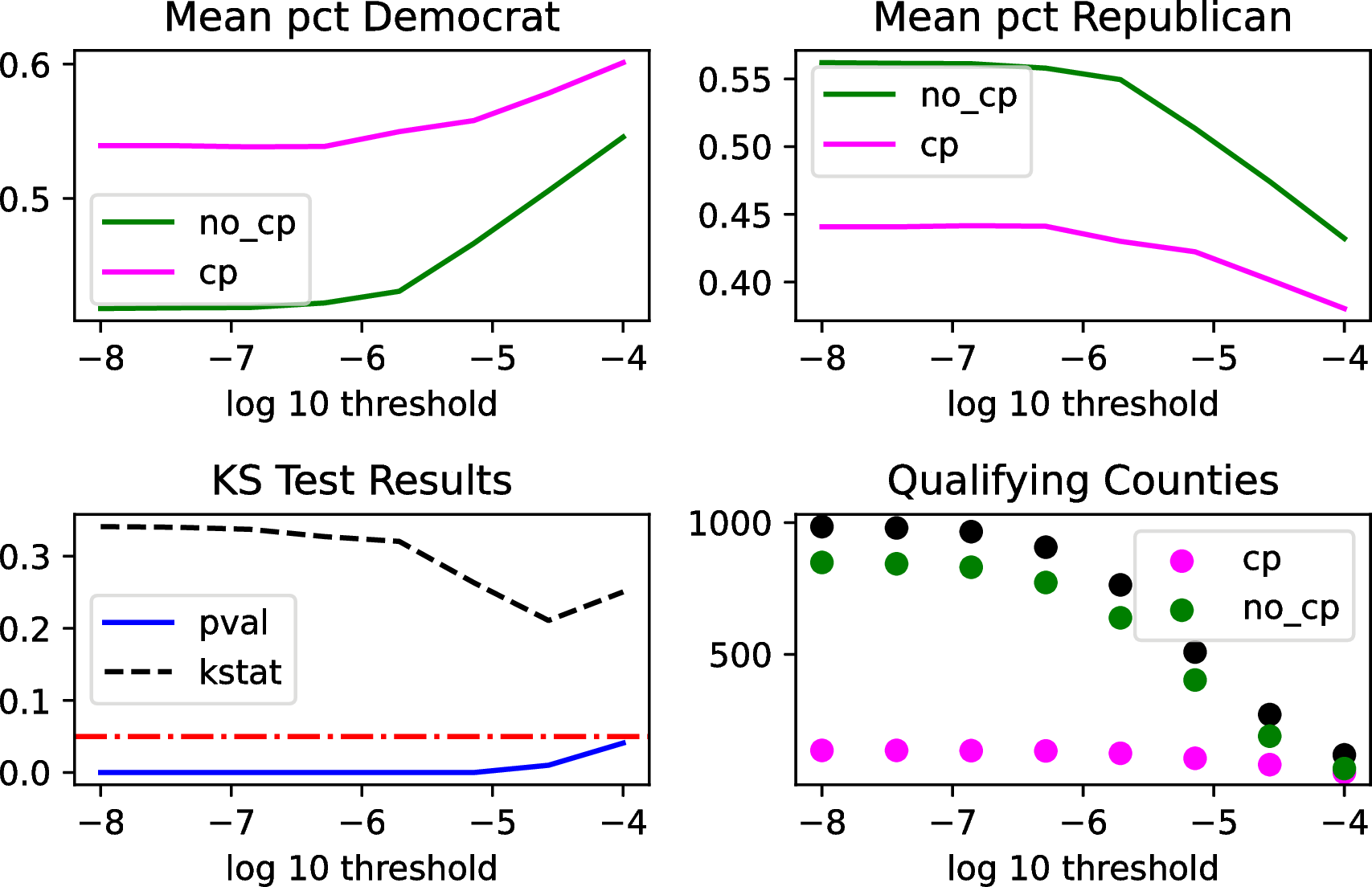

Supplement: S1 Fig — Top: The mean percent having voted Democrat(left) and Republican (right) in the 2020 Presidential election of the counties with and without change points as the threshold is increased at the log 10 scale. When the threshold is between -8 and -6 the gap in mean vote share between counties with and without abrupt park visitation changes is stable. As the threshold increases past -6 the gap begins to shrink, with the counties with abrupt changes becoming slightly more democrat, and the counties without abrupt changes becoming much more democrat, and both becoming less Republican. Bottom Left: The p-value (blue) and statistic(black dashed) results of the KS 2 sample test on the partisan differences in counties with and without abrupt changes as the threshold increases. The pvalue is stable until the threshold is greater than -5, when it begins to increase, but never crosses the p = 0.05 significance threshold (red dashed). The k statistic remains stable until the threshold is increased past -6, when it decreases, but never falls below 0.2. Bottom Right: The number of counties (black) which meet inclusion criteria as the visitation threshold is increased. There is is rapid decline in counties included in the study beginning at a threshold of -6. Past a threshold of -5 fewer than half of all counties in our data set meet inclusion criteria, and at -4 there are almost none. The number of counties with an abrupt visitation change (pink) remains constant in the study until a threshold greater than -5, reflecting that these are among the counties with the greatest visitation. The number of counties without a change (green) declines almost in parallel to the total (black) counties, indicating that the threshold criteria eliminates these counties almost exclusively. (TIF) [file pgph.0000766.s001.tif]

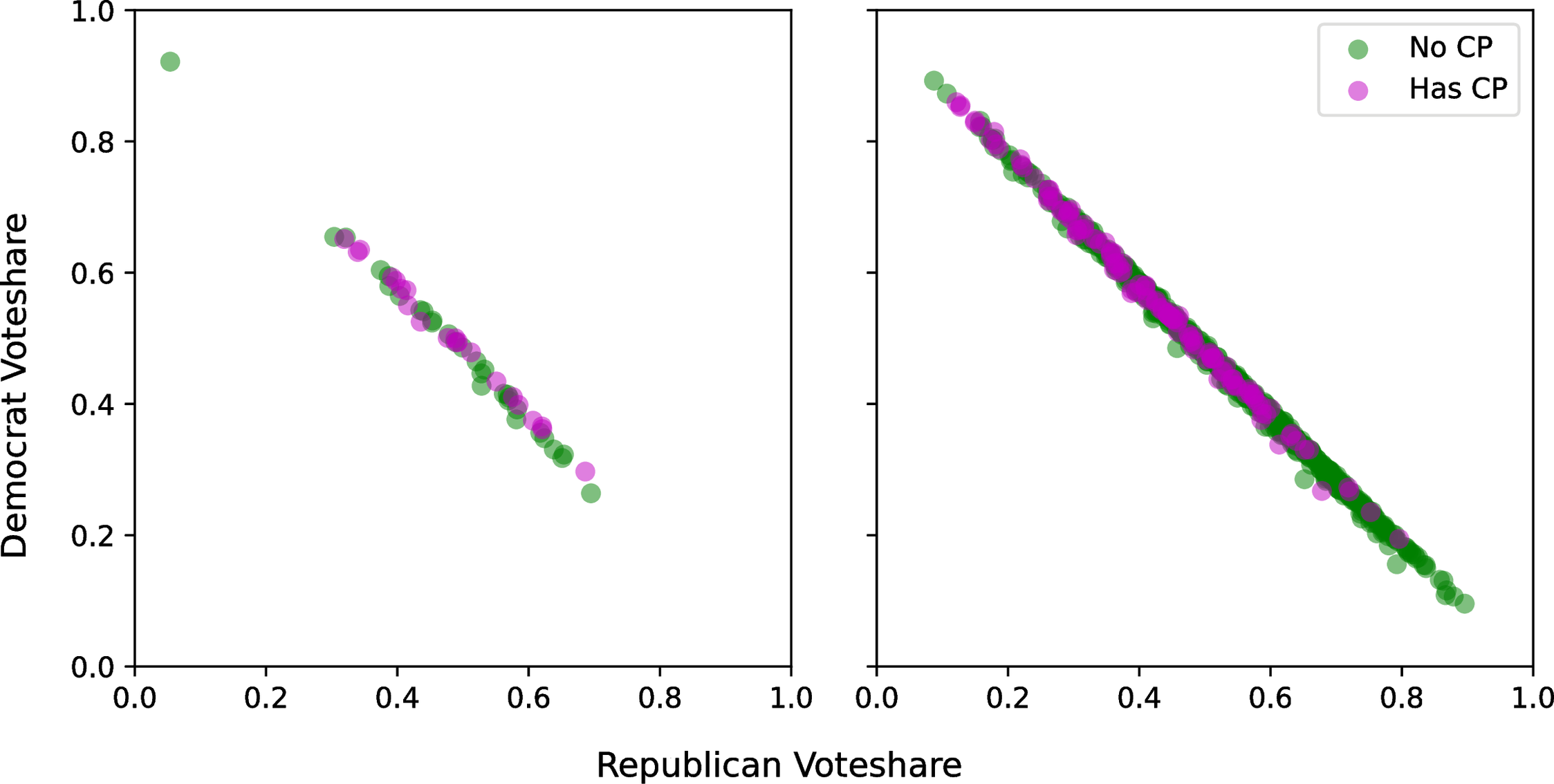

Supplement: S2 Fig — The location in the x-y plane is determined by the percent of votes for the Republican(x) and Democratic(y) candidates in the 2020 Presidential Election. (TIF) [file pgph.0000766.s002.tif]
