# Supplementary material for: Spatial changes in park visitation at the onset of the pandemic
Source: PLOS Glob Public Health. 2022 Sep 13;2(9):e0000766. doi: 10.1371/journal.pgph.0000766 (PMC10021367; doi:10.1371/journal.pgph.0000766)
Supplement: Attachment — Submitted filename: rebuttal_letter.pdf [file pgph.0000766.s003.pdf]

Reply to Reviews

`Submission:` PGPH-D-22-00198
`Manuscript:` Spatial changes in park visitation at the onset of the pandemic

We thank the reviewers for giving us the opportunity to revise and improve our manuscript. Below you'll find responses to each comment.

Reviewer 1

Comment

The structure of the article seems to be more along the lines of those submitted to engineering and computer science conference proceedings (section I, II etc). Is this format in line with the journal?

We are willing to restructure the paper if the editor believes it is appropriate.

Comment

Park visitation estimates is likely underestimated (since it solely relies on mobile data) - this is not reported as a limitation

We thank the reviewer for their keen observation and have added the following acknowledgement of this limitation to the manuscript:

" The park visitation estimates used in this study are necessarily underestimates because they rely on mobile devices, and do not account for multiple visits made by a single device in one day. Other measurements of visitation could improve accuracy of visitation estimates."

Comment

Fig. 1: Visitation for 2019 is plotted in blue - correct to Visitation for 2019 is plotted in green.

We thank the reviewer for noticing this discrepancy, and have updated the manuscript to correct it.

Comment

In the discussion section, it would be useful to comment on how the abrupt changes in park visitation in certain sections could have affected their health and well-being. In fact, for a journal that focuses on health, it would be ideal to include some health outcomes as well in their analysis with respect to changes in park visitation.

We are reluctant to speculate on the health implications of our observations given the limitations of the data. However, we outline the relevancy to public health in the introduction and allude to potential beneficial health outcomes.

Comment

With regards to the statistical analysis, I feel I do not have the necessary technical expertise to evaluate the methods. Given the strong dependence of the results of this article on the advanced statistical methods used, I would recommend that this paper is reviewed by a statistician as well.

We thank the reviewer for their commitment to a rigorous review process, and believe that the comments made by Reviewer 3 indicate our methods have been assessed by a qualified scientist.

Reviewer 2

Comment

Author has address the the question well defined manner. He has explained all section very well. Author has defined the methods properly, shared the data link. From my side there is no comment. We should go ahead with the publication.

We thank the reviewer for their favorable comments!

Reviewer 3

Comment

To summarize, this study presents a fairly comprehensive analysis of data relating to spatial and temporal changes in park visitations, aggregated at different spatial levels. The manuscript is well written in general. The comments below are from the perspective of statistical rigor of the article.

We thank the reviewer for their appreciation of our writing, and for the insights they provide regarding our communication of our methods in the following comments.

Comment

1) While BEAST is chosen as the main analysis vehicle, the authors rely on the KS test to reveal critical findings. This is somewhat a jarring mix and match between frequentist and Bayesian methods. At the very least, this should be acknowledged and clarified in the methods, that after time series analysis and decoupling, the authors chose simpler empirical methods (KS test) for comparisons.

Thank you for your attention to detail, and for providing the opportunity to further discuss our methodology. In this study we were challenged to identify regions in which a notable change in behavior occurred, and then to compare these regions. The results of the Bayesian inference we initially apply to the time series of park visitations are used as a classifier. The frequentist method of KS testing is employed as a way of comparing the two types of classification. We agree that these are very different approaches, and have striven to demonstrate that they serve to address two separate problems, with two different types of data (times series in one case, and distributions in the other).

We have added a paragraph to the beginning of the Methods section for the purposes of clarifying the roles that the two methods play in the over arching study design:

"In this study we compare regions in the United States where abrupt non-seasonal changes in park visitation did and did not occur at the onset of the pandemic. In order to make these comparisons we begin by classifying regions as having or not having an abrupt change point by applying a change point detection algorithm to aggregated time series of park visitation in each of the regions. Once regions have been classified, the distributions of the regions across population, income, share of employment by industry, and voteshare in the 2020 presidential election are compared."

Comment

2) The authors rely on a fair amount of manual wrangling and judgement calls to choose the parameters of the Beast application - in terms of profile and number of change points and other settings. Was robustness examined in terms of other settings, such as say, 2 change points?

Thank you for your interest in our efforts! In the preliminary stage of our research we examined the performance of BEAST on our set of time series under a variety of settings. Manual inspection revealed that the algorithm was consistent in identifying the change point at the onset of the pandemic for the regions where it was found in the paper.

When given leeway to identify multiple change points, the algorithm surfaced a few other regions to have a pandemic changepoint - though on manual inspection it was only after one or more other change points had been selected first, and these changepoints were much less convincing than those that were found when only a single changepoint was alotted. It was apparent that the inclusion of extra change points was allowing overfitting.

We chose to qualify an abrupt change point having occurred at the onset of the pandemic if it was the most substantial change identified by BEAST during the year. We argue this is a defensible choice, as messaging to "stay at home" was strongest at this time.

Comment

3) Details of the Beast application (length and number of MCMC chains chosen), as well as characteristics of the MCMC convergence should be commented upon.

We thank the reviewer for their diligence. It has come to our attention that there exists a more widely known version of BEAST which was published in a 2007 paper by Drummond and Rambaut. We believe there may be some confusion and so we have added a few lines to our supplementary materials for disambiguation about the method used, and have included more details of the BEAST application. The added text is provided below.

" BEAST was used in a non-traditional manner as a classifier in this work. The algorithm referred to is The Bayesian Estimator for Abrupt and Seasonal Changem which is developed in the 2019 paper by Zhao et al. [?], and is available as a package known as RBeast for the language R through CRAN. This disambiguation is provided due to the existence of a separate and more widely known BEAST algorithm developed by Drummond and Rambaut in 2007 [?].

In our application of BEAST we chose 3 MCMC chains each with a length of 10,000 samples."

Comment

4) Spatial correlation may confound some of the findings - since the time series reported and not ”independent” sequences of observations. This should at least be acknowledged in the findings and future work should consider spatio-temporal methods.

We thank the reviewer for their keen consideration. In this work park visitations are aggregated to either a county or state level. It is our belief that aggregations at this spatial scale will minimize spatial correlation because the people visiting parks in one region are unlikely to have traveled the distance to visit a park in a different region. We agree that future work would benefit from consideration of potential correlation, especially if a more granular spatial scale is applied, and so have included the following line in our limitations and future work section:

”Future work in this direction could benefit from spatio-temporal methods, which would account for any spatial correlation of park visits.”
